# Supplementary material for: Keeping the power on to home medical devices
Source: PLoS One. 2020 Jul 9;15(7):e0235068. doi: 10.1371/journal.pone.0235068 (PMC7347141; doi:10.1371/journal.pone.0235068)
Supplement: S1 Data — (DOCX) [file pone.0235068.s001.docx]

Dear PLOS ONE Team

The data repository for the paper is at <https://doi.org/10.14264/uql.2020.802>

Thank you

Richard Bean
