# Supplementary material for: Keeping the power on to home medical devices
Source: PLoS One. 2020 Jul 9;15(7):e0235068. doi: 10.1371/journal.pone.0235068 (PMC7347141; doi:10.1371/journal.pone.0235068)
Supplement: Attachment — Submitted filename: hmed-response-to-comments-10-apr-2020.docx [file pone.0235068.s002.docx]

**We thank the reviewers for their remarks regarding our manuscript and have improved our submission as appropriate. We have detailed our responses to the reviewers’ remarks below.**

**Reviewer #1:** The novelty of this paper if any have not been significant. Substantial scientific aspects are missing in the paper such as the design of the proposed algorithm and hardware of the system. My recommendation is the paper is not suitable to publish in PLOS ONE (rejected).

***In our paper, we present a novel method for unsupervised clustering of electricity phases for keeping home medical devices connected to the grid. We have better emphasised the novelty of the clustering approach relative to existing attempts (Pezeshki and Wolf) on p.6. This paper was submitted to a digital health technology call and keeping digital health technology powered underpins the efficacy of such technologies. Since the focus of the paper is on digital health, we have described sufficient details of our algorithm on pages 6-8 for the purpose of the application to digital health. We clarified on page 6 that we are in the process of investigating a patent for the algorithm and therefore cannot disclose further details, however the accuracy relative to best practice (Pezeshki and Wolf) has been elaborated (page 6).***

***The (Ouija Lite) hardware that we used is a commercial product of Redback Technologies. To better outline the design of the hardware as requested by Reviewer 1; we have provided an overview of the system and how it functions within the cloud environment. However further details of the hardware (e.g. PCB design) is not relevant to this paper where the focus is on home medical devices.***

**Reviewer #2:** This article addressed the power supply issue regarding home medical devices which has practical values. The work can be of certain reference in the area. I would like to recommend consider it for acceptance after further improvement.

Here are some of my impression about the work:

1. The present study appears some what too engineering which needs fruther in-depth improvement especially on fundamental parts.

***We are unsure what the reviewer is referring to in terms of “too engineering [focused?]”, but we are happy to make any specific and appropriate suggested changes.***

2. Some earlier literatures on mobile health care are needed.

***We have added the following references to mobile health care. If we have missed any other key references, we are happy to add these.***

***Jones, K. R., Lekhak, N., & Kaewluang, N. (2014). Using mobile phones and short message service to deliver self‐management interventions for chronic conditions: A meta‐review. Worldviews on Evidence‐Based Nursing, 11(2), 81-88.***

***Aref-Adib, G., McCloud, T., Ross, J., O'Hanlon, P., Appleton, V., Rowe, S., ... & Lobban, F. (2019). Factors affecting implementation of digital health interventions for people with psychosis or bipolar disorder, and their family and friends: a systematic review. The Lancet Psychiatry, 6(3), 257-266.***

***O’Neil, A., Cocker, F., Rarau, P., Baptista, S., Cassimatis, M., Barr Taylor, C., ... & Oldenburg, B. (2017). Using digital interventions to improve the cardiometabolic health of populations: a meta-review of reporting quality. Journal of the American Medical Informatics Association, 24(4), 867-879.***

3. Originality of the curent work is not very clear. Power is a necessity and tremendous devices are available for such needs.

***The novelty of the work is in providing a method for unsupervised, as opposed to supervised clustering of phases. We amended the text of our manuscript to clarify this and increase the emphasis on this in the document. We have also added a paragraph to describe the difference between unsupervised and supervised clustering of phases for clarity. While there may be UPS devices to mitigate the effects of power outages, they typically don’t have a backup battery suitable or sufficient to power home medical devices. Detecting the phase and correctly and ensuring that HMED users are not cut off saves lives.***

***One example: We have changed the following text:***

***The new algorithm is unsupervised and does not require access to transformer level voltage data.***

***To:***

***The new algorithm is unsupervised and does not require access to transformer level voltage data, as the supervised approach of Pezeshki and Wolf (2012) does. Instead, voltage time series data is collected at each of the houses for the purpose of the analysis.***

4. Adding some more basic innovative theoretical strategies will enhance curent work.

***We are unsure what the reviewer is referring to in terms of “basic innovative theoretical strategies”, but we are happy to make any specific and appropriate suggested changes.***

5. Unique feature of current energy supply perhaps can still be distinguished with existing one.

***We are unsure what this comment is referring to. We speculate that the reviewer may have misunderstood that three-phase energy supplies already exist. Properties are connected to different phases and energy networks don’t have reliable information with regards to the phase that a domestic home where a HMED device is in use might be. Consequently, the need to detect the phase is critical to keeping the power connected. If the reviewer can clarify what they mean, we are happy to make any specific and appropriate suggested changes.***

**Reviewer #3:** The authors present what appears to be a very novel idea with useful application. However, the development and operation of the phase algorithm, at least as it's written here is seems vague. In general, a more thorough presentation of the algorithm and analysis in reference to the actual operator measurements are needed. Please include some calculations and/or code on how, for example the error rates and plots were obtained. Figures 2 and 4 should be clearer/labeled better.

***We thank the reviewer for their remarks. We have added a diagram to clarify how the phase detection algorithm is employed for the application, with a voltage time series plot of three houses to explain how the correlation coefficient is calculated between pairs of voltage time series.***

It would be most convincing to provide a map of the houses sampled, where the algorithm's phase is displayed, as well as the actual phase based on operator records. Authors should provide a copy of the operator records, perhaps as supplemental data.

***We have added some example calculations, changed the figure labels on figures 2 and 4, and provided an example map. We will provide the supplemental and operator record data as Supplementary Data in a repository after the paper is accepted for publication.***

***We added the sentence “This is the best of the six possible phase orderings - values from each row and column are chosen so that all rows and columns are used, and the sum of the values is maximized.” to clarify the process of error measurement.***

***We have added a coloured map with street names removed to indicate the relative positions of the houses and the transformers.***

***We will provide R code on how the error rates and plots were calculated – in the repository.***
