# Supplementary material for: Keeping the power on to home medical devices
Source: PLoS One. 2020 Jul 9;15(7):e0235068. doi: 10.1371/journal.pone.0235068 (PMC7347141; doi:10.1371/journal.pone.0235068)
Supplement: Attachment — Submitted filename: hmed-response-to-comments-1-jun-2020.docx [file pone.0235068.s003.docx]

**We thank the reviewers for their remarks regarding our manuscript and have improved our submission as appropriate. We have detailed our responses to the reviewers’ remarks below.**

**Reviewer #2 and #3 comments have been made in the previous version.**

**Dr Al-Ameri commented**

*Following our decision letter, please try to address reviewers comments on the original submission. If you can clarify the novelty of your manuscript by introducing more details about the methodology as requested by reviewer #1. One of our reviewers has expected explanation for the following sentence in the revised manuscript; “In our paper, we present a novel method for unsupervised clustering of electricity phases for keeping home medical devices connected to the grid”*

*Please explain to reviewer #1why you cannot disclose further detail!.*

*Thank you for submitting your manuscript to PLOS ONE.*

**The following comment from the response to comments addresses this request for explanation.**

Reviewer 1 is actually **incorrectly** stating that details about the hardware (and in fact the algorithm) are missing as we have stated which commercial hardware we are using and we have disclosed how the algorithm works (on pages 6-8) just not a full disclosure and this is because there is a patent pending – there are a number of pages of detail. What would the editor recommend we do to address a factually incorrect comment from reviewer 1?

**Reviewer #1:** The novelty of this paper if any have not been significant. Substantial scientific aspects are missing in the paper such as the design of the proposed algorithm and hardware of the system. My recommendation is the paper is not suitable to publish in PLOS ONE (rejected).

***In our paper, we present a novel method for unsupervised clustering of electricity phases for keeping home medical devices connected to the grid. We have better emphasised the novelty of the clustering approach relative to existing attempts (Pezeshki and Wolf) on p.6. This paper was submitted to a digital health technology call and keeping digital health technology powered underpins the efficacy of such technologies. Since the focus of the paper is on digital health, we have described sufficient details of our algorithm on pages 6-8 for the purpose of the application to digital health. We clarified on page 6 that we are in the process of investigating a patent for the algorithm and therefore cannot disclose further details, however the accuracy relative to best practice (Pezeshki and Wolf) has been elaborated (page 6).***

***The (Ouija Lite) hardware that we used is a commercial product of Redback Technologies. To better outline the design of the hardware as requested by Reviewer 1; we have provided an overview of the system and how it functions within the cloud environment. However further details of the hardware (e.g. PCB design) is not relevant to this paper where the focus is on home medical devices.***
